# Number of Days Required to Estimate Habitual Activity Using Wrist-Worn GENEActiv Accelerometer: A Cross-Sectional Study

**DOI:** 10.1371/journal.pone.0109913

**Published:** 2016-05-05

**Authors:** Christina B. Dillon, Anthony P. Fitzgerald, Patricia M. Kearney, Ivan J. Perry, Kirsten L. Rennie, Robert Kozarski, Catherine M. Phillips

**Affiliations:** 1 HRB Centre for Diet and Health Research, Department of Epidemiology and Public Health, University College Cork, Cork, Ireland; 2 Department of Epidemiology & Public Health, University College Cork, Western Gateway Building, Western Road, Ireland; 3 Department of Statistics, University College Cork, Western Gateway Building, Western Road, Ireland; 4 School of Life and Medical Sciences, University of Hertfordshire, Hatfield, Hertfordshire, AL10 9AB, United Kingdom; Vanderbilt University, UNITED STATES

## Abstract

**Introduction:**

Objective methods like accelerometers are feasible for large studies and may quantify variability in day-to-day physical activity better than self-report. The variability between days suggests that day of the week cannot be ignored in the design and analysis of physical activity studies. The purpose of this paper is to investigate the optimal number of days needed to obtain reliable estimates of weekly habitual physical activity using the wrist-worn GENEActiv accelerometer.

**Methods:**

Data are from a subsample of the Mitchelstown cohort; 475 (44.6% males; mean aged 59.6±5.5 years) middle-aged Irish adults. Participants wore the wrist GENEActiv accelerometer for 7-consecutive days. Data were collected at 100Hz and summarised into a signal magnitude vector using 60s epochs. Each time interval was categorised according to intensity based on validated cut-offs. Spearman pairwise correlations determined the association between days of the week. Repeated measures ANOVA examined differences in average minutes across days. Intraclass correlations examined the proportion of variability between days, and Spearman-Brown formula estimated intra-class reliability coefficient associated with combinations of 1–7 days.

**Results:**

Three hundred and ninety-seven adults (59.7±5.5yrs) had valid accelerometer data. Overall, men were most sedentary on weekends while women spent more time in sedentary behaviour on Sunday through Tuesday. Post hoc analysis found sedentary behaviour and light activity levels on Sunday to differ to all other days in the week. Analysis revealed greater than 1 day monitoring is necessary to achieve acceptable reliability. Monitoring frame duration for reliable estimates varied across intensity categories, (sedentary (3 days), light (2 days), moderate (2 days) and vigorous activity (6 days) and MVPA (2 days)).

**Conclusion:**

These findings provide knowledge into the behavioural variability in weekly activity patterns of middle-aged adults. Since Sunday differed from all other days in the week this suggests that day of the week cannot be overlooked in the design and analysis of physical activity studies and thus should be included in the study monitoring frames. Collectively our data suggest that six days monitoring, inclusive of Saturday and Sunday, are needed to reliably capture weekly habitual activity in all activity intensities using the wrist-worn GENEActiv accelerometer.

## Background

Accurately measuring habitual physical activity is crucial to understanding the relationship between frequency, duration, type and amount of physical activity and health. A range of subjective and objective methods to quantify physical activity and sedentary behaviour exist. Objective measures such as accelerometers and pedometers provide information on patterns of physical behaviour within a given day and across several days, are feasible for large studies, are less prone to error and no recall is necessary. Thus in comparison to subjective methods, objective measures provide significantly more reliable data on habitual physical behaviour.

Physical behaviour is influenced by a range of factors including demographic characteristics, emotional influences and behavioural attributes [1]. As a result patterns of physical behaviour show substantial intra- and inter-individual variation, the extent of which plays a major role on data quality and reliability [2]. Methodological issues such as duration of monitoring-frame, position of wear, accelerometer type and wear-time compliance may also affect data quality. Modern devices are fully waterproof and can be worn on the wrist, resulting in improved wear-time compliance as the device can be worn all day and does not need to be removed for water based activities [3, 4]. Minimising the number of days monitoring will likely have important implications on wear-time compliance. Extended monitoring periods can be a burden to participants and financially costly, leading to the removal of the device, reduced wear-time, and subsequently reduced data quality. Thus a current challenge is determining an appropriate monitoring frame for researchers who want to minimise participant burden and maximise wear-time compliance.

Several studies have examined a suitable monitoring frame to accurately measure physical behaviour in adults [5–8]. These studies have varied in terms of statistical analysis, position of wear, type of accelerometer and time-frame of interest, producing variable monitoring frames of 7 days, 5 days, 5–6 days and 3–5 days, respectively [5–8]. In addition, some examined the appropriate monitoring frames to reliably estimate habitual physical behaviour intensities individually [5, 6]. Matthews et al. (2002) concluded that 3–4 days monitoring were required to correctly measure moderate-to-vigorous physical activity (MVPA), and that 7 days were needed to reliably estimate physical inactivity [5]. Findings by Scheers et al. (2012) recommended overall that both Saturday and Sunday in addition to at least 3 weekdays were needed to obtain reliable estimates of habitual physical activity [6]. Hart et al. (2011) recommended 5, 3 and 2 days monitoring for sedentary behaviour, light activity, moderate and vigorous activity respectively [8]. Such conflicting recommendations highlight the need to determine an appropriate monitoring frame to reliably measure both habitual physical activity and sedentary behaviour for each accelerometer and activity intensity. Importantly no studies to date have sought to determine a suitable monitoring frame to accurately measure physical behaviour using the wrist-worn GENEActiv accelerometer. Only this wear location was examined. Compliance will vary across wear locations. The GENEActiv accelerometer is a waterproof device which can be worn 24 hours a day for long durations (up to 30 days), on multiple different bodily positions (hip, thigh, waist and wrist). The wrist-worn position has been found to provide equally accurate data to hip and waist mounted devices and are associated with better compliance to wear protocols [3, 4, 9]. Furthermore, the 24 hour wear protocol implemented in this paper may have important implications on calculation of daily physical behaviour estimates as errors associated with missing data imputation (due to device removal) will be avoided and additionally waterproofing will allow water-based activities of higher intensity activities to be captured. The GENEActiv accelerometer is a relatively new device in the field of habitual physical activity research. Unlike many other accelerometers, data is collected and stored as raw acceleration in *g* units (m/s^2^) for offline analysis thereby allowing a range of data processing techniques to be applied post data-collection.

Thus the aim of this study is to examine the intra- and inter-individual variability across days, and thus identify an appropriate monitoring frame for capturing weekly habitual physical behaviour in middle-aged adults using the wrist-worn GENEActiv accelerometer.

## Methods

### Subjects

A population representative random sample (Mitchelstown cohort) was recruited from a large primary care centre in Mitchelstown, County Cork, Ireland [10]. The primary care centre includes 8 general practitioners and the practice serves a catchment area of approximately 20, 000 with a mix of urban and rural residents. Participants were randomly selected from all registered attending patients in the 50–69 year age group. In total 3, 807 potential participants were selected from the practice list. Following exclusion of duplicates, deaths and ineligibles, 3, 043 were invited to participate in the study and of these 2, 047 (49.2% male) completed the questionnaire and physical examination components of the baseline assessment (response rate 67%) during the study period (April 2010 and May 2011). Accelerometers were introduced into the study in January 2011. Of the 745 cohort participants seen between January and May of 2011, 475 (44.6% males; mean aged 59.6±5.5 years) subjects agreed to participate (response rate 64%) and of these 397 (46.1% males; mean 59.6±5.5years) had valid accelerometer data.

### Ethics Statement

The study was approved by the Clinical Research Ethics Committee of University College Cork. Written informed consent to participate was obtained from all participants.

### Accelerometer protocol

The GENEActiv accelerometer was introduced in the latter half of the study. Objective physical activity levels were assessed using a tri-axial, GENEActiv accelerometer. The accelerometer (ActivInsights Ltd, Kimbolton, Cambridgeshire, United Kingdom) comprised a tri-axial STMicroelectronics accelerometer with a dynamic range of +/-8 g (1 g = 9.81 m/s^2^), where g represents gravitational unit, and was attached to the participants’ preferred wrist with a strap. The technical reliability and validity of this accelerometer has been reported elsewhere [11]. For the current study, acceleration was sampled at 100Hz and the accelerometer worn for 7-consecutive days. Following return of the accelerometer to the co-ordination centre, the data was extracted using GENEActiv software and then collapsed using the following, sum of the vector magnitude, equation ($\sum |\sqrt{x^{2}+y^{2}+z^{2}}-\;g|$) [11]. This equation is used to calculate the sum (∑) of the signal magnitude vector $\sqrt{x^{2}+y^{2}+z^{2}}$ with gravity subtracted (-g). The sum is calculated for a specific time interval (epoch) e.g. 60 second epoch. Each time interval, from the daytime wear-time (6am-12am) periods, was categorised based on validated cut-off points for dominant and non-dominant wrist wear. Cut-point values were created from a convenience sample of 56 volunteers aged between 18 and 65 years, free from injury and in good health. Data was sampled at a frequency of 30Hz and collapsed into 15-second epochs. Oxygen consumption (VO^2^) was measured using a portable metabolic unit (Cosmed K4B^2^, Rome, Italy) [12]. Activities (sitting, standing, dish washing, floor sweeping, slow walking, fast walking and jogging) were completed in ascending intensity. Participants were asked to complete each activity at a pace that was comfortable to them, but within each speed range: slow walking (2.5–4.5 km/hour), brisk walking (4.5–6.5 km/hour) and light jogging (6.5–8.5 km/hour). Intensity cut-points (sum of the vector magnitude counts) created for dominant and non-dominant wrist-wear based on all activities, with the exception of dish washing, are presented in Table 1.

**Table 1:** Sensitivity, specificity, AUC and GENEActiv cut-points based on 3 and 6 METS.

Intensity*	Sensitivity	Specificity	Area under the curve (95%CI)	GENEActiv cut-points (SVMgs (15s epoch))	
**Dominant wrist**				**30Hz**	**100Hz**
Sedentary	91.6	92.4	0.97	<57.5	<191.8
Light	NA	NA	NA	57.5–84.3	191.8–281.5
Moderate	67.9	67.9	0.694	84.4–178.4	281.6–595
Vigorous	97.1	97.0	0.994	>178.4	>595
**Non-dominant wrist**					
Sedentary	92.9	92.4	0.98	<47.5	<158.5
Light	NA	NA	NA	47.5–78.3	158.5–261.8
Moderate	70.5	68.6	0.716	78.4–148.5	261.9–495
Vigorous	97.1	97.0	0.992	>148.5	>495

* sedentary (<1.5 METS), light (1.5–2.99 METS), moderate (3.00–5.99 METS), vigorous (>6 METS)

NA; not applicable as sedentary and moderate intensity cut-points provide the margins for light intensity

Wear and non-wear time was identified by the procedure outlined by Van Hees et al., (2011) [13]. Non-wear time was calculated for each accelerometer axis on the basis of the standard deviation (SD) and the value range. The procedure was carried out on successive blocks of 30 minutes. A block was categorised as non-wear time if the SD was less than 3.0 mg (1 mg = 0.00981 m.s^-2^) or if the value range was less than 50 mg, for at least two out of the three axes. Four-hundred and seventy-five subjects wore the accelerometer. One-hundred and sixty-six participants wore the GENEActiv accelerometer on their non-dominant wrist, 210 wore the device on their dominant hand while 21 did not have this data recorded. Choice of wrist was selected by the participant to ensure comfort and wear compliance, this decreased the likelihood of the participant removing the device and thus increased the quality and quantity of data for analysis as fewer participants were deemed invalid (<7 days wear) for analysis. Following exclusion of 16 participants with missing data due to technical issues and 62 participants with less than 10 hours of wear time activity during daytime hours on any day, the remaining 397 subjects were eligible for further analysis. The number of participants with various numbers of valid days (days in which the participant recorded >10 hours of wear time data) of data are presented in Table 2.

**Table 2:** Number of participants with valid days (>10 hours of wear time) of data.

Number of valid days wear	Number of participants
**7 days**	397
**6 days**	27
**5 days**	12
**4 days**	4
**3 days**	3
**2 days**	4
**1 days**	6
**0 days**	6

### Statistical analysis

Analysis was performed separately for each intensity category. Individual median and 25^th^ and 75^th^ percentiles for minutes spent in each activity category were calculated for each day, data non-normally distributed. Data are reported as median and 25^th^ and 75^th^ percentiles unless otherwise stated. Kruskal-Wallis p-values assessed whether activity levels varied significantly across days of the week. Spearman pairwise correlations determined the association between days of the week. Number of days required to reliably estimate habitual physical activity was assessed using repeated measures analysis of variance (ANOVA), intra-class correlations (ICC), and modified Spearman-Brown formula [14]. Repeated measures ANOVA established whether minutes spent in activity differed across days. In the case of the violation of the assumption of sphericity, the Greenhouse-Geisser adjusted F was interpreted. If an overall significant F level was shown, post hoc tests (Tukey HSD pairwise comparisons) were used to assess differences between days. Effect size was assessed to determine the amount of variation in the criterion (total weekly minutes) that was accounted for by various days of monitoring. Coefficient of variation ((SD/mean)*100) was calculated to explain intra-individual and inter-individual variability. Intra-individual variability was calculated for each individual using weekly days of data while inter-individual variability was analysed as the group mean and SD for weekly minutes. ICCs were calculated to determine the reliability of using any single day of activity to estimate daily activity using 7 days of data. An ICC of 0.80 is considered standard to designated acceptable reliability [2]. A modified version of the Spearman-Brown calculation determined the intraclass reliability coefficient associated with 1, 2, 3, 4, 5, 6, and 7 days of activity [5, 14, 15]. The intraclass reliability coefficient was estimated as the proportion of total variance attributable to between-subject variance as follows: *[(between-subject variation)* ^*2*^ */ ((between-subject variation)*c ^*2*^ *+ ((within-subject variation)* ^*2*^ */n))]*, where *n* is the number of days monitoring. All statistical analyses were conducted using Stata (version 12, Stata Corp, College Station, Texas, USA), except coefficient of variation and Spearman-Brown formula which were performed by hand. An alpha level of 0.05 was set to evaluate significance.

## Results

### Descriptive analysis

Median time (minutes) spent in each activity type across days of the week was calculated separately for men and women (Table 3). Overall, differences in median activity levels across days were significant (P<0.05), with the exception of vigorous activity (P>0.05). Among all subjects time spent in sedentary activity was greatest on Sunday (946 minutes). Among men, sedentary activity was higher on Sunday (956 minutes) compared to all other weekdays (889–912 minutes), while women were most sedentary on Sunday through Tuesday (930–940 minutes). Both men and women were more physically active on weekdays. Time spent in vigorous activity was similarly low for men and women throughout the week.

**Table 3:** Daily duration (minutes) of sedentary, light, moderate and vigorous activity.

**Total (n = 397)**					
	**Sedentary**	**Light**	**Moderate**	**Vigorous**	**MVPA**
**Monday**	926 (833, 984)	94 (64, 140)	50 (24, 93)	1 (0, 5)	56 (25, 100)
**Tuesday**	921 (837, 981)	98 (64, 135)	48 (24, 91)	1 (0, 6)	52 (25, 101)
**Wednesday**	911 (829, 976)	100 (68, 143)	55 (25, 95)	1 (0, 5)	59 (27, 100)
**Thursday**	908 (842, 977)	106 (66, 140)	56 (26, 93)	1 (0, 5)	58 (26, 100)
**Friday**	903 (826, 977)	106 (68, 148)	57 (25, 100)	1 (0, 5)	62 (25, 106)
**Saturday**	910 (839, 989)	100 (65, 142)	51 (23, 98)	1 (0, 5)	56 (23, 103)
**Sunday**	946 (872, 1004)	77 (48, 117)	42 (17, 82)	0 (0, 3)	46 (18, 91)
**p-value**	<0.001	<0.001	<0.001	0.81	<0.001
**Men (n = 183)**					
	**Sedentary**	**Light**	**Moderate**	**Vigorous**	**MVPA**
**Monday**	909 (798, 972)	98 (66, 154)	60 (29, 116)	1 (0, 7)	69 (29, 126)
**Tuesday**	906 (813, 979)	103 (62, 146)	61 (25, 112)	1 (0, 7)	66 (27, 120)
**Wednesday**	903 (802, 970)	99 (67, 156)	66 (32, 118)	1 (0, 6)	76 (33, 122)
**Thursday**	901 (815, 978)	104 (66, 143)	66 (31, 107)	1 (0, 5)	71 (31, 116)
**Friday**	889 (802, 977)	100 (66, 156)	65 (29, 111)	1 (0, 6)	74 (30, 121)
**Saturday**	912 (840, 987)	99 (65, 135)	58 (25, 99)	1 (0, 6)	64 (25, 103)
**Sunday**	956 (878, 1009)	70 (43, 108)	45 (18, 85)	1 (0, 4)	47 (19, 85)
**p-value**	<0.001	<0.001	0.009	0.77	0.01
**Women (n = 214)**					
	**Sedentary**	**Light**	**Moderate**	**Vigorous**	**MVPA**
**Monday**	931 (869, 990)	92 (58, 133)	40 (22, 76)	1 (0, 3)	46 (22, 82)
**Tuesday**	930 (867, 985)	97 (66, 130)	43 (21, 75)	1 (0, 5)	47 (22, 84)
**Wednesday**	918 (850, 980)	100 (68, 135)	49 (23, 83)	1 (0, 4)	52 (25, 87)
**Thursday**	915 (854, 974)	107 (68, 138)	48 (23, 83)	1 (0, 4)	51 (24, 88)
**Friday**	910 (844, 979)	110 (68, 146)	48 (23, 84)	1 (0, 4)	52 (25, 89)
**Saturday**	909 (834, 990)	103 (67, 148)	46 (21, 94)	1 (0, 4)	51 (22, 104)
**Sunday**	940 (862, 1002)	89 (51, 124)	38 (16, 80)	0 (0, 3)	43 (16, 85)
**p-value**	<0.001	<0.001	<0.001	0.84	<0.001

Data is presented as median (25th, 75th percentile). P-values presented as Kruskal-Wallis, tests the difference in median activity levels across days of the week.

### Pairwise comparisons

All Spearman pairwise correlations between days of the week were significant (*P*<0.001). The range of pairwise correlations varied across days of the week and intensity type; sedentary (0.59–0.79), light (0.59–0.77), moderate (0.59–0.77), vigorous (0.37–0.60) and MVPA (0.58–0.78), (S1 Table). The mean pairwise correlations across days of the week were 0.72, 0.68, 0.69 and 0.41 for sedentary, light, moderate and vigorous activity respectively.

### Variance analysis

There were significant differences between days for sedentary (*P*<0.01), light activity (P<0.01), moderate activity (*P*<0.01) and MVPA (P<0.01), whereas vigorous activity (*P =* 0.15) was not significantly different between days. In relation to sedentary and light activity, Sunday differed from all other days of the week (*P*<0.05). The differences in mean minutes between days was small; sedentary (0.7%) moderate (0.4%), vigorous (0.2%) and MVPA (0.4%), except for light activity (1.6%). The mean intra-individual variability for each activity type were; sedentary (30.3%), light activity (45.4%), moderate activity (60.8%), vigorous activity (73.7%) and MVPA (61.6%), while inter-individual variability was 1.8–178% of total variance; sedentary (3.3%), light activity (2.3%), moderate activity (1.8%), vigorous activity (107%) and MVPA (178%).

### Intra-class reliability coefficients

**Fig 1:**
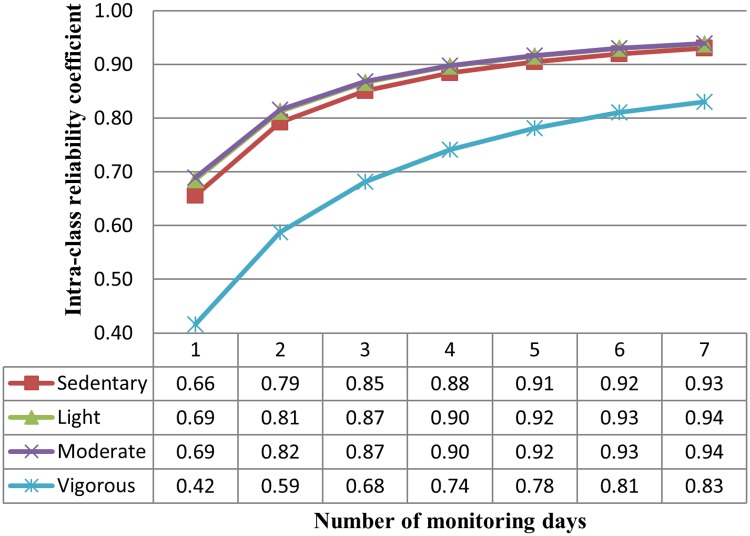
Reliability coefficient for number of days monitoring. Fig 1 illustrates the reliability coefficient associated with different length monitoring frames. The results propose that between 59–82%, 68–87%, 74–90% and 78–92% of variance was explained for by using 2 days, 3 days, 4 days and 5 days monitoring to represent 7 days of habitual activity.

The ICC for any single day for sedentary, light, moderate activity, vigorous activity and MVPA was calculated, 0.66, 0.69, 0.69, 0.42 and 0.68 respectively. These results demonstrate that between 42–69% variance was accounted for using any single day of data collection to represent 7-day habitual activity. When ICC was calculated by gender, ICC did not alter, with the exception of vigorous activity (32% and 46% for men and women respectively). Spearman-Brown Formula calculated reliability coefficients for combination of days (Fig 1). These results indicate that between 59–82%, 68–87%, 74–90% and 78–92% of the variance was accounted for using 2 days, 3 days, 4 days and 5 days monitoring to represent 7 day habitual activity. The appropriate monitoring frames for each intensity of activity are 3 days, 2 days and 6 days for sedentary behaviour, light and moderate activity and MVPA and vigorous activity respectively. All remaining combinations were higher than 0.80.

## Discussion

Our results indicate that the number of monitoring days required to estimate weekly habitual activity vary according to physical behaviour intensity. Based on our findings, we recommend that data collection periods should be based on activity intensity; sedentary (3 days), light activity (2 days), moderate activity (2 days), vigorous activity (6 days) and MVPA (2 days). Because variability between activity intensities across days of the week was small any combination of days appears to be sufficient to acquire a stable weekly estimate of physical activity and sedentary behaviour. Our findings support current guidelines recommending inclusion of both weekend and week days in physical behaviour monitoring frames. Many large studies (e.g. NHANES and Biobank) using similar protocols may apply our findings to reduce monitoring time-frames and increase device turnover in the field. Additionally our result could influence the analysis of these studies, i.e. if moderate activity is the exposure of interest a minimum wear period of 2 days (inclusive of one weekend day) can be implemented in turn decreasing the number of days and or person excluded from analysis and thus increasing the power to finding significant associations with health outcomes.

While this is the first study to examine the required number of monitoring days needed to accurately measure physical behaviour in adults using the wrist-worn GENEActiv accelerometer, other accelerometers have been examined in this context [5–8]. Overall these studies vary in terms of statistical analysis, position of wear, type of accelerometer and time-frame of interest, resulting in variable monitoring frames of 7 days, 5 days, 5–6 days and 3–5 days, respectively [5–8]. All studies utilised different accelerometers; Compute Science Applications (CSA) accelerometer [5], SenseWear Armband [6], Caltrac accelerometer [7] and the ActiGraph [8], and positioned the device on multiple body positions; the hip [5], arm [6] and waist [7, 8]. Several assessed the appropriate monitoring frames to reliably estimate habitual physical behaviour intensities independently [5, 6, 8]. Matthews et al. (2002) determined that 3–4 days monitoring were required to accurately measure MVPA [5]. Scheers et al. (2012) suggested that both Saturday and Sunday and at least 3 weekdays were needed to obtain reliable estimates of habitual physical activity, and only 3 days data collection was needed to capture light activity [6]. Hart et al. (2011) proposed monitoring frames individually for sedentary behaviour, light activity, moderate and vigorous activity; 5, 3 and 2 days monitoring respectively [8]. These inconsistent recommendations emphasise the need to establish an appropriate monitoring frame to reliably capture habitual physical behaviour for each accelerometer, activity intensity and position of wear.

Our results add to the current literature by reporting the number of monitoring days needed to reliably estimate habitual physical behaviour using GENEActiv accelerometers. The number of days needed to reliably estimate habitual physical behaviour vary according to activity intensity and statistical tests used [2]. Tudor-Locke et al. (2005) contend that no single statistical test is considered adequate to fully understand the issues underlying the calculation of an appropriate monitoring frame [16]. As recommended by Tudor-Locke et al. (2005), which employed a wide range of statistical techniques to determine number of days needed for an appropriate monitoring frame, Spearman-Brown prophecy formula has been used in the majority of studies investigating appropriate monitoring frames [5, 14, 17]. Results of Spearman-Brown calculations and ICC for a single day identified consistent monitoring frames for all activity intensities (>1 day monitoring).

In addition, the moderate to high pairwise correlations across days indicated a clear tendency for activity patterns to be consistent across the days, with the exception of vigorous activity where correlations were low suggesting that vigorous activity patterns varied throughout the week, thus explaining the longer monitoring frame. In terms of sedentary and light activity, Sunday had the lowest correlations, suggesting the activity patterns on Sunday are less consistent with other days in the week. Greater between-subject variation and lesser within-subject variation across days results in shorter monitoring frames. Light and moderate intensity activities have the shortest monitoring frames; this could be due to higher levels of between-subject variation and lower levels of within-subject variation across days of the week compared to sedentary and vigorous activity, and thus 2 days of monitoring is sufficient to capture variation in light and moderate intensity activities. In addition, light and moderate intensity activities are more likely to include household activities and activities such as exercise which tend to be planned, structured and repetitive [18]. The same could be said for vigorous activity, however due to the very low levels of vigorous activity measured in this population, variation between- and within-subjects would be hard to capture accurately, thus resulting in a larger monitoring frame. This is supported by the low pairwise correlations across days, which indicate inconsistent activity patterns across the days.

### Study strengths and limitations

A main strength of our study is the use of a valid and reliable activity monitor which is capable of assessing time spent in sedentary, light, moderate and vigorous activity categories [11]. In addition, this accelerometer collects data as raw acceleration and stores the data as g units for offline analysis thereby allowing for efficient data cleaning, management of spurious data, and the application of various known data processing algorithms post-data collection. Further strengths include the 24-hour study protocol, the high study participation rate and large sample size. Notwithstanding these strengths one limitation of this study is that we only examined the required number of monitoring days needed to reliably estimate weekly habitual activity. Further investigation could be expanded into how many days/weeks of monitoring represent a month, a season, or a year of habitual activity using the wrist-worn GENEActiv accelerometer. Kang et al. (2009) examined a suitable monitoring frame to capture year-round averages of pedometer measured physical activity and found 5 consecutive days and 6 random days to be necessary [19]. In addition, many studies have reported seasonal and monthly variations in physical activity leading to recommendations for physical behaviour data collection to occur during certain seasons and specific months of the year [20–22]. Generalizability of our findings may also be limited. The Mitchelstown cohort was a random sample of middle-aged adults, 50–69 years of age, in an area which was representative of both urban and rural population in Ireland. The sub-sample of the Mitchelstown cohort for whom accelerometer data was collected, differed by gender, in that women were more likely to agree to wear the accelerometer. In addition, participants were recruited from a primary care centre, and therefore could have more health problems or be more health conscious. However it should be noted that there were no statistically significant differences in age, gender, education or BMI between those included and excluded in the final analysis.

The data for this study was collected over 7 consecutive days at a frequency of 100Hz and collapsed into 60s epochs. Under these conditions our results demonstrate the number of monitoring days required to reliably assess weekly habitual activity for each type of intensity. We observed marked differences between weekdays, Saturday and Sundays. If the outcome of interest, for further studies, involves a more detailed examination of patterns of activity both Saturday and Sunday should be included in the monitoring frame. Similarly our gender specific findings, such as comparatively high sedentary activity in women on Monday, should be considered. This consideration may be particularly pertinent when examining overweight and obese adults whose activity on weekend days has been shown to be particularly distinct from normal weight subjects across week days [23, 24].

## Conclusion

This study examined the number of monitoring days needed to accurately estimate habitual physical activity and sedentary behaviour from the wrist-worn GENEActiv accelerometer in middle-aged adults. Our data indicates 6 days monitoring are required to reliably capture weekly activity in all activity categories. These findings may have important implications in terms of study design and data reduction strategies. Further study protocols employing our recommendations may benefit from reduced number of data collection and processing days and associated reductions in person-time and study cost.

## Supporting Information

S1 Table Spearman pairwise correlation coefficient of physical activity intensity by days of week. (PDF)
