# Supplementary material for: Number of Days Required to Estimate Habitual Activity Using Wrist-Worn GENEActiv Accelerometer: A Cross-Sectional Study
Source: PLoS One. 2016 May 5;11(5):e0109913. doi: 10.1371/journal.pone.0109913 (PMC4858250; doi:10.1371/journal.pone.0109913)
Supplement: S1 Table — (PDF) [file pone.0109913.s001.pdf]

**S1 Table: Spearman pairwise correlation coefficient of physical activity intensity by days of week.**							
	**Sunday**	**Monday**	**Tuesday**	**Wednesday**	**Thursday**	**Friday**	**Saturday**
**Sedentary behaviour**							
Sunday	**1.00**						
Monday	0.59	**1.00**					
Tuesday	0.63	0.73	**1.00**				
Wednesday	0.64	0.74	0.73	**1.00**			
Thursday	0.63	0.72	0.75	0.77	**1.00**		
Friday	0.68	0.72	0.73	0.75	0.79	**1.00**	
Saturday	0.71	0.67	0.66	0.69	0.71	0.71	**1.00**
**Light activity**							
Sunday	**1.00**						
Monday	0.59	**1.00**					
Tuesday	0.62	0.70	**1.00**				
Wednesday	0.65	0.71	0.77	**1.00**			
Thursday	0.60	0.67	0.71	0.74	**1.00**		
Friday	0.64	0.66	0.70	0.74	0.74	**1.00**	
Saturday	0.68	0.67	0.66	0.70	0.69	0.72	**1.00**
**Moderate activity**							
Sunday	**1.00**						
Monday	0.59	**1.00**					
Tuesday	0.59	0.69	**1.00**				
Wednesday	0.63	0.71	0.69	**1.00**			
Thursday	0.64	0.73	0.75	0.76	**1.00**		

Friday	0.68	0.72	0.71	0.72	0.77	**1.00**	
Saturday	0.70	0.67	0.64	0.64	0.67	0.68	**1.00**
**Vigorous activity**							
Sunday	**1.00**						
Monday	0.46	**1.00**					
Tuesday	0.45	0.60	**1.00**				
Wednesday	0.37	0.50	0.55	**1.00**			
Thursday	0.43	0.52	0.47	0.53	**1.00**		
Friday	0.52	0.53	0.51	0.50	0.53	**1.00**	
Saturday	0.48	0.49	0.40	0.43	0.47	0.48	**1.00**
**MVPA**							
Sunday	**1.00**						
Monday	0.58	**1.00**					
Tuesday	0.60	0.70	**1.00**				
Wednesday	0.62	0.73	0.68	**1.00**			
Thursday	0.65	0.74	0.75	0.76	**1.00**		
Friday	0.68	0.72	0.72	0.72	0.78	**1.00**	
Saturday	0.69	0.67	0.64	0.64	0.68	0.68	**1.00**

All values have a significance <0.001
